# Physiology of the subconscious: Autonomic activation during sexual dreaming

**DOI:** 10.1016/j.sleepx.2026.100174

**Published:** 2026-01-17

**Authors:** Emmanuel Eroumé A Egom, Bernadette Sandrine Lema, Elijah-Bill Christopher Nguem Nguem

**Affiliations:** a Hartford HealthCare Heart & Vascular Institute, Hartford Hospital, Hartford, CT, USA; b Institut du Savoir Montfort (ISM), Ottawa, ON, Canada; c Institute of Medical Research and Medicinal Plants Studies (IMPM), Yaoundé, Cameroon; d Heaven Foundation / Fondation CIEL, Hartford, CT, USA; e Harmony Health Physical and Spiritual Wellness Center (Harmony Care), Hartford, CT, USA

**Keywords:** Sexual dreams, Autonomic-like experiences, Sleep, Self-reported physiology, Dreaming

## Abstract

**Objective:**

To evaluate self-reported autonomic-like symptoms following sexual dreams in the SLEEP Study and characterize their perceived physiological patterns.

**Methods:**

In a cross-sectional online study, 301 female-identifying adults reported physical and emotional sensations experienced immediately after sexual dreams. We summarized symptom prevalence using descriptive statistics.

**Results:**

Increased heart rate (57.4 %) and sweating (35.0 %) were most frequently reported, followed by anxiety (33.9 %) and muscle tension (23.0 %). A minority (20.8 %) reported no symptoms, indicating variability in perceived arousal or recall. Symptom patterns reflected common co-occurrence of self-reported cardiovascular-like and affective experiences.

**Discussion:**

These findings suggest that sexual dreams are often accompanied by self-reported autonomic-like experiences, although these reports do not represent objective physiological measurements. This brief report isolates the symptom dimension of the SLEEP Study dataset—a component not analyzed in our prior *Sleep Research* publication—and highlights the potential value of self-reported responses for studying REM-linked emotional arousal.

## Highlights

• Sexual dreams often elicit autonomic-like experiences during sleep.
• Increased heart rate was the most commonly reported symptom.
• A minority reported no symptoms, showing variability in perceived arousal.
• This brief report isolates physiological-like symptoms not analyzed previously.
• Findings highlight the need for objective sleep-physiology validation.

## 1 Introduction

Dreams are a window into emotion–autonomic coupling during sleep [1,2]. While sexual dreams are common, their **physiological** signatures are rarely quantified outside laboratory settings. In this study, a ‘sexual dream’ refers to any dream involving erotic activity, romantic-sensual interaction, or emotionally intimate content with a sexual component. Building on our Sleep Research article [3], we summarize autonomic-like symptoms reported immediately after sexual dreams in a community cohort.

## 2 Methods

### 2.1 Study design and participants

This brief report represents a focused secondary analysis of autonomic-like symptoms within the SLEEP Study dataset. The broader psychological findings were previously published in *Sleep Research* [3]; those analyses did not examine autonomic-like symptoms as independent physiological outcomes. The SLEEP Study is a cross-sectional, survey-based investigation conducted by Heaven Foundation/Fondation CIEL with anonymized data handling and electronic consent. Adults (N = 301) residing in the United States completed an online questionnaire on dream experiences and post-dream symptoms. The analytic sample included 301 female-identifying adults (90 % identifying as women, 10 % nonbinary), with a mean age of 39.5 years (SD = 12.3; range 19–104). Control comparisons (e.g., nightmares or neutral dreams) were not included because the approved survey focused exclusively on sexual-dream experiences. This brief report therefore describes autonomic-like symptoms within this specific dream category only.

### 2.2 Measures

Participants indicated post-dream increased heart rate, sweating, muscle tension, anxiety, none, or other (single/multiple selections allowed).

Post-dream symptoms were assessed with the item:

After this dream, did you experience any of the following?

• Increased heart rate
• Sweating
• Anxiety
• Muscle tension
• None
• Other (free text)

Participants could select multiple responses.

### 2.3 Analytic approach

We summarized symptom prevalence using descriptive statistics (proportions with 95 % confidence intervals). Analyses focused on descriptive characterization and co-occurrence patterns of reported cardiovascular-like and affective symptoms. No inferential or correlational analyses were performed, consistent with the hypothesis-generating and self-reported nature of the dataset.

## 3 Results

**Fig. 1:**
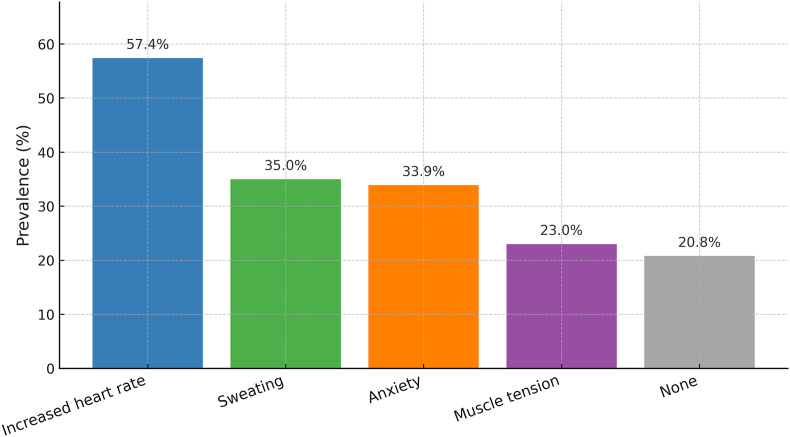
**Prevalence of post-dream symptoms following sexual dreams (N=301).** Bars show % of participants endorsing each symptom: increased heart rate (57.4 %), sweating (35.0 %), anxiety (33.9 %), muscle tension (23.0 %), and none (20.8 %). Labels denote percentages.

Increased heart rate was most frequent (57.4 %), followed by sweating (35.0 %) and anxiety (33.9 %). Muscle tension occurred in 22.95 % of respondents, while 20.8 % reported **no** symptoms. These distributions are illustrated in Fig. 1 and detailed with 95 % confidence intervals in Table 1. These distributions describe the prevalence and co-occurrence of self-reported post-dream physical and emotional symptoms, as summarized in Table 1 and Fig. 1.

**Table 1:** Prevalence of post-dream symptoms with 95 % Wilson confidence intervals (N = 301).

Symptom	n	Prevalence (%)	95 % CI lower (%)	95 % CI upper (%)
Increased heart rate	173	57.4	51.8	62.9
Sweating	105	35.0	29.7	40.4
Anxiety	102	33.9	28.8	39.4
Muscle tension	69	23.0	18.5	28.0
None	63	20.8	16.7	25.9

Values are prevalence (%) with 95 % **Wilson** confidence intervals, computed from counts rounded from study percentages (N = 301).

## 4 Discussion

Self-reported post-dream symptoms indicate that sexual dreams are commonly associated with perceived autonomic-like experiences, reflecting subjective emotional and bodily arousal reported by participants. The co-occurrence of cardiovascular-like (heart rate, sweating) and affective (anxiety) symptoms is consistent with parallel subjective arousal experiences. The ∼21 % asymptomatic subgroup may reflect lower perceived arousal, variability in recall, or differences in reporting style.

These self-reported autonomic-like experiences reflect **perceived arousal states** and do not constitute objective evidence of autonomic or sleep-stage–specific physiological activation. The asymptomatic subgroup may reflect lower perceived arousal, variability in recall, or differences in reporting style.

Clinically, dream-elicited self-reports may complement PSG-based markers by offering an ecologically valid, low-burden subjective index of emotional arousal during sleep, without implying specific sleep-stage mechanisms. Future work should integrate ambulatory physiology (e.g., PPG-derived pulse, EDA) with experience sampling to validate these patterns against objective signals.

This brief report is limited by its female-only sample, reliance on subjective recall, lack of dream-type control conditions, and absence of objective physiological recordings. These constraints reflect the structure of the original SLEEP Study instrument.

## 5 Conclusion

Sexual dreaming is frequently accompanied by self-reported autonomic-like experiences and may serve as a non-invasive, hypothesis-generating probe of subjective emotion–bodily coupling during sleep.

## CRediT authorship contribution statement

**Emmanuel Eroumé A Egom:** Writing – review & editing, Writing – original draft, Visualization, Supervision, Methodology, Investigation, Formal analysis, Data curation, Conceptualization. **Bernadette Sandrine Lema:** Writing – review & editing, Writing – original draft, Supervision, Methodology, Investigation, Formal analysis, Data curation, Conceptualization. **Elijah-Bill Christopher Nguem Nguem:** Writing – review & editing, Writing – original draft, Formal analysis, Data curation, Conceptualization.

## Ethics statement

All participants provided electronic informed consent; the study was non-interventional and conducted in accordance with the Declaration of Helsinki.

## Declaration of generative AI use

No generative AI tools were used to generate scientific content or images. Language polishing tools were limited to grammar and style checks; authors take full responsibility for the content.

## Funding

No external funding was received.

## Declaration of competing interest

The authors declare that they have no known competing financial interests or personal relationships that could have appeared to influence the work reported in this paper.

## Data Availability

De-identified data are available from the corresponding author on reasonable request.
